# Performance and Durability of Chalcedonite Reactive Powder Concrete

**DOI:** 10.3390/ma18184258

**Published:** 2025-09-11

**Authors:** Joanna Julia Sokołowska, Piotr Woyciechowski, Szymon Żerek

**Affiliations:** 1 Department of Building Materials Engineering, Institute of Building Engineering, Faculty of Civil Engineering, Warsaw University of Technology, 00-637 Warsaw, Poland; piotr.woyciechowski@pw.edu.pl; 2 Faculty of Civil Engineering, Warsaw University of Technology, 00-637 Warsaw, Poland

**Keywords:** chalcedonite, reactive powder concrete, high-strength concrete, high-performance concrete, concrete, steam curing, heat treatment, durability, frost resistance, carbonation

## Abstract

The objective of this study was to evaluate the technical properties and assess the durability of a novel high-performance concrete with aggregates composed entirely of reactive powders derived from chalcedonite—a mineral previously not utilized in HPC technology. Since there is insufficient information on chalcedonite-based concretes in the scientific literature, the presented research aims to address these knowledge gaps. The characterization of the chalcedonite powder involved the determination of specific gravity, particle size distribution, specific surface area, and particle morphology through microscopic analysis. The hardened chalcedonite-based and reference quartz-based high-performance concretes were subjected to a comprehensive suite of tests to determine their physical properties (bulk density, water absorption, and capillary absorption) and mechanical properties (flexural and compressive strength). Durability was further assessed based on compressive strength criteria, including frost resistance and carbonation resistance. To simulate long-term performance and better evaluate the durability of the high-performance concretes, specimens were tested following standard water curing and after additional maturation processes, including thermal treatment, which in the extreme case resulted in a seven-day compressive strength of 176.9 MPa, a value higher by 56.7 MPa (corresponding to an increase of 47.1%) compared to the strength of the identical concrete not subjected to thermal treatment. To explore the potential for architectural applications, particularly in outdoor environments, capillary absorption testing was of particular importance, as it provided insight into the material’s resistance to eventual pigment leaching from the mineral matrix.

## 1. Introduction and Research Significance

While there is a lot of concern about concrete’s high environmental and carbon footprint [1,2], this composite is still one of the most widely used construction materials, serving both structural and decorative purposes. Over time, concrete has undergone continuous development to enhance its performance characteristics, including its mechanical strength [3] after [4,5]. Advancements in concrete technology have led to the development of reactive powder concrete (RPC) [6], a material that, while structurally similar to conventional concrete, is produced by refining aggregate size, increasing cement content, and minimizing water usage [7]. Thus, RPC and ordinary concrete (OC) represent two distinct types of cementitious composites, differing in aggregate materials and grading, aggregate design approach, and setting mechanisms, including reactive aggregate interactions beyond cement hydration. RPC represents a relatively recent innovation (the earliest known structure made from RPC is a pedestrian/bicycle bridge in Sherbrooke, Canada, constructed in 1997 [8]) and remains under active development [9]. Currently, this material is used, for example, to increase the strength of beams and columns [10,11,12], or slabs [13], because of its exceptional mechanical performance, which is several times greater than that of standard concrete. Reactive powder concrete, based on its compressive strength, can be categorized into very-high-performance concrete (VHPC), with strengths ranging from 100 to 150 MPa, or ultra-high-performance concrete (UHPC), characterized by compressive strengths exceeding 150 MPa [14]. Using an appropriate particle size distribution of fillers (which usually do not exceed 2 mm in size) and heat treatment (autoclaving or steam curing) or/and reinforcing with steel fibers, RPC can reach compressive strengths of 200–500 MPa [14,15,16]. And RPC with a compressive strength of 200 MPa obtains tensile strength in the range of 30–60 MPa, a Young’s modulus in the range of 50–60 GPa, fracture energy between 20 and 40 kJ/m^2^, and an ultimate tensile strain of 0.005–0.007 [6,11].

Despite obvious advantages over traditional concrete in terms of mechanical properties, RPC is not yet widely adopted due to several limitations, including the following:

- A lack of standardized testing procedures and design codes (which poses significant challenges for research and practical application);
- Difficulties in producing the mix (improper mixing can lead to excessive air entrainment, compromising strength);
- Complex rheology issues (e.g., high early-age drying shrinkage and autogenous shrinkage, or creep, etc.);
- Limited possibilities of using equipment for pumping and laying the standard concrete mix;
- High production and material cost dependent on the limited availability of suitable raw materials; The last limitation encourages the search for new components that could be used to produce RPCs. The authors see the possibility of using chalcedonite as a valuable aggregate for this type of composite. This approach is a novelty as the chalcedonite mineral has not been utilized in reactive powder concrete technology yet.

## 2. Chalcedonite as the Unique Rock

### 2.1. Chalcedonite’s Origin

**Figure 1:**
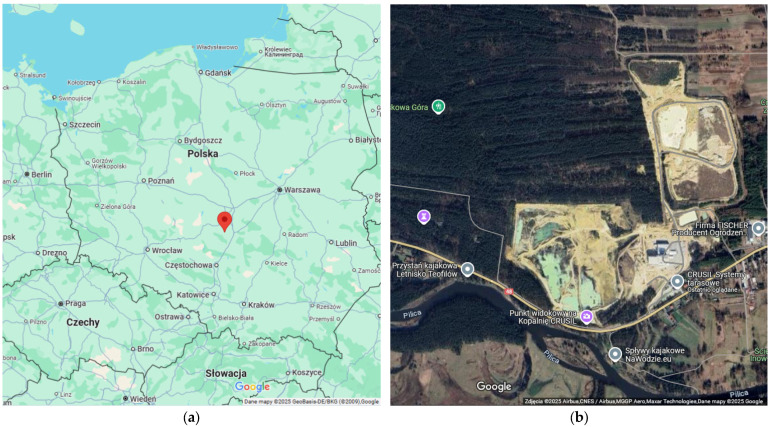
Location of the Teofilów deposits of the unique rock, chalcedonite: (**a**) location in Poland [Maps data: ©2025 GeoBasis-DE/BKG (© 2009), Google]; (**b**) view of the quarries [Maps data: ©2025 Airbus, CNES/Airbus, MGGP Aero, Maxar Technologies, Maps data: ©2025 Google].

Chalcedonite is a siliceous sedimentary rock classified as a unique geological material due to its limited geographical occurrence. It is found in the deposits of Dęborzynka, Gąbinin, Lubocz, and Teofilów (Figure 1), all located on the Rawska Plateau in the vicinity of Tomaszów Mazowiecki and Nowe Miasto, Poland. Among these, only the Teofilów deposit is currently documented and actively exploited [17].

Due to its high content of siliceous sponges and chalcedony, it is referred to as spongiolite chalcedonite [18,19]. Two theories describe the formation of this unique mineral. The first theory assumes that it was formed as a result of the secondary silicification of Jurassic limestone-marl formations in a tropical climate during the pre-Miocene period [20]. The second theory assumes that chalcedonite was formed through the dissolution, precipitation, and crystallization of silica derived from dead organisms that lived in a warm and shallow open sea [18]. Nonetheless, the primary mineral component of chalcedonite is chalcedony, accompanied by minor quantities of quartz, opal, iron hydroxides, pyrite, manganese compounds, and clay minerals [17,18,19,20,21].

### 2.2. Characterization of Teofilów Chalcedonite Deposit

The Teofilów deposit of chalcedonite covers an area of 577,437 m^2^, with estimated geological reserves amounting to approximately 21.587 million tonnes. The concentration of silica within the deposit is uneven; therefore, chalcedonite occurs in the deposit in the form of beds and locally discontinuous layers, which are interbedded with chalcedonite rubble mixed with clay, silt, and siliceous pelite [17]. The volumetric mineral composition of chalcedonite is as follows: chalcedony, opal, and authigenic quartz constitute 68.3–95.4%; detrital quartz and other terrigenous components account for 0.3–6.6%; and free or infilled pores represent 2.0–24.7% [19]. Silicified organic remains are abundant in chalcedonite and include fragments of mollusk shells, brachiopods, foraminifera, echinoid plates, and numerous sponge spicules [18,19]. Chalcedonite fractions collected in the sedimentation tanks of the Inowłódz mine and used in water treatment and wastewater purification technologies showed a similar mineral composition: chalcedony and authigenic quartz, 81.6–88.6%; terrigenous quartz, 3.6–14.6%; feldspars, 0.3–1.7%; and clay rock fragments, 2.0–8.5% [22]. Since the chalcedonite varies in terms of its petrographic composition and porosity, variability in the physical and mechanical properties can also be found [18]. For example, specific gravity obtains values within the range of 2500–2700 kg/m^3^. Excavated materials’ specific surface area ranges from 3 to 6 m^2^/g. The mesoporous structure is characterized by high pore uniformity, with a total pore volume of 0.03–0.04 cm^3^/g [17]. In general, chalcedonite occurs in the Teofilów deposits in two main varieties [18,19,20,21] with the third variety occurring marginally [23]:

- Milky-blue variety—compact, homogeneous, and hard, with a splintery and uneven fracture; upon impact, it breaks into sharp-edged, flat fragments; it contains cavernous depressions with loose infillings.
- Gray variety—significantly less compact, with higher porosity and a greater content of clay minerals. Its fracture surface resembles pumice, containing pores that partially reflect the shapes of decayed organisms. The color may transition to yellow or rusty hues, depending on the variable content of iron compounds.
- Yellow-brown-red variety—characterized by high porosity and a substantial amount of iron oxides, which impart its distinctive coloration.
- In the research, the second variety of chalcedonite was used.

### 2.3. Chalcedonite’s Current Applications

Due to its limited distribution and multifunctional properties, chalcedonite is considered a rare and valuable raw material. Owing to its mesoporous structure and developed external surface area, chalcedonite is widely used in water treatment technologies, particularly as an efficient filtration medium [22,23,24,25]. Its effectiveness in removing manganese and iron from water is evidenced by the shallow depth of the iron removal zone within the filter bed, the rapid onset of manganese (II) removal, and favorable hydraulic properties that enable a high filtration capacity and extended operational cycles [24,25]. Furthermore, chalcedonite-based filter beds have demonstrated the effective removal of ammonia nitrogen through nitrification processes in wastewater treatment. Its sorptive properties also make it a promising candidate for the remediation of oil spills [17].

When it comes to its use as a building material, chalcedonite initially was used only as a road base material. But the chalcedonite coarse aggregates presented insufficient strength for road construction, and such a material solution was not justified economically [23]. Starting from the 1990s, chalcedonite has found broader applications as a filler in paints, varnishes, enamelware, sealants, and putties [17,26]. There were also trials to use chalcedonite as a silica-bearing additive in cement production [27], an additive in cement pastes and concretes [28,29], a binder in geopolymers [30], or as a filler in biodegradable polymer composites [31,32]. Due to its porosity and water absorption (of c.a. 14% [33]), it is also well-suited for use in drainage—e.g., roofing granules or soil substrates, where it helps maintain consistent moisture levels. This study investigates a novel application of chalcedonite—as an aggregate in reactive powder concrete, RPC.

## 3. Materials and Methods

**Figure 2:**
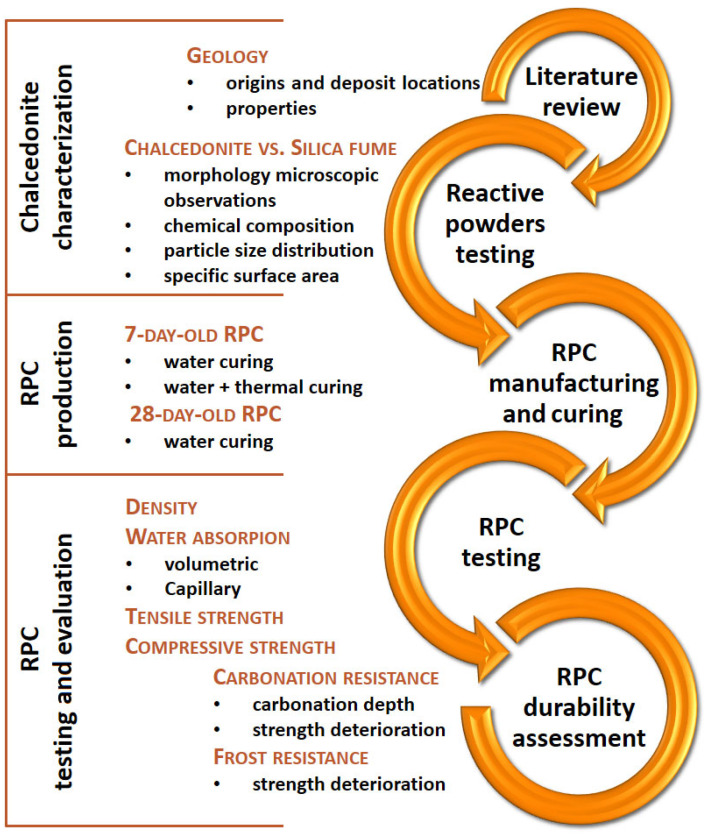
Scheme of the research program of the presented study.

The research presented in this paper followed a multi-stage approach, beginning with a comprehensive characterization of chalcedonite. This included a literature review—covering a few older sources that are rarely updated due to the uniqueness of this rock—and original experimental studies on chalcedonite powders. These studies involved microscopic observations of grain morphology, chemical composition determination, and particle size distribution and specific surface area characterization, benchmarked against a quartz filler from the reference composite. Subsequently, innovative concrete mixes were designed and produced, incorporating a variant with alternative thermal curing, to evaluate their fundamental physical and mechanical properties as well as their durability performance. The research program, which integrates both novel material characterization and performance assessment, is illustrated in Figure 2.

### 3.1. Qualitative and Qualitative Composition of Tested Composites

The subjects of the research were reactive powder concretes with quartz and silica fume aggregates (grading up to 0.5 mm) and RPC where the aggregate was completely substituted with chalcedonite fillers (also grading up to 0.5 mm). In both cases, white Portland cement was used to provide the light color of the fresh mixes and hardened composites. The components included the following:

- White Portland cement of high early strength, CEM I 52.5R (Aalborg Portland Holding A/S, Aalborg Øst, Denmark) meeting the requirements of EN 197-1:2011 [34] and EN 196-1:2016 [35] European standards;
- Quartz sand of fraction 250/500 µm (Kwarcmix, Tomaszów Mazowiecki, Poland);
- Quartz powder of fraction 0/120 µm (Quarzwerke GmbH, Frechen, Germany);
- Chalcedonite fillers of fraction 0/250 µm and fraction 250/500 µm (CRUSIL, Inowłódz, Poland);
- White silica fume class G94, the byproduct of zirconium dioxide production with the content of (SiO_2_ + ZrO_2_) ≥ 94% (wt.) and specific surface area ≥ 15 m^2^/g, fulfilling the requirements of ASTM C 1240 [36] (Mikrosilika Trade, Stalowa Wola, Poland);
- Tap water meeting the requirements of the EN 1008:2002 European standard [37];
- Super-plasticizing admixture on the basis of polycarboxylate ether (Sika Poland Sp. z o.o., Warsaw, Poland).

The detailed characteristics of white Portland cement according to the manufacturer’s specification are given in Table 1. The experimentally determined characteristics of aggregates, including chalcedonite fillers, are provided in the next section.

Four reactive powder concrete compositions were designed—two (marked REF and NREF) with regular quartz sand, quartz powder and silica fume and two (marked CH and NCH) that contained only chalcedonite aggregates. The research, however, included six variants, because the two selected were duplicated in order to apply a different method of curing in conditions of elevated temperature, hence the composite designations additionally include REF^1^ and CH^1^—i.e., subjected to thermal treatment. The quantitative compositions of all tested concretes are listed in Table 2.

### 3.2. Characteristics of the Quartz and Chalcedonite Aggregates

The tested chalcedonite materials were sourced from Teofilów deposits located on the Rawska Plateau in the vicinity of Tomaszów Mazowiecki and Nowe Miasto, Poland (Figure 1a). They were collected, ground and fractioned into fractions of 0/250 µm (0/0.25 mm) and 250/500 µm (0.25/0.50 mm) by the CRUSIL company. The chalcedonite aggregates were then subjected to additional tests to determine more detailed characteristics for the purposes of the research presented.

The chemical composition of the chalcedonite provided by the manufacturer [38] and according to Kosk [22] is given in Table 3. The same manufacturer’s specification also mentions a specific gravity of 2600 kg/m^3^, grain porosity up to 30%, absorbability within the range of 4–10%, abrasiveness (determined in the Micro-Deval testing machine) between 6 and 15% and compressive strength (of the chalcedonite rock) between 60 and 120 MPa. Taking into account that the specific gravity of chalcedonite, a rock rich in silica, is very close to pure silica fume and pure quartz specific gravity (2650 kg/m^3^), the mass replacement of quartz aggregates (sand and powder) with chalcedonite aggregates is generally justified.

**Figure 3:**
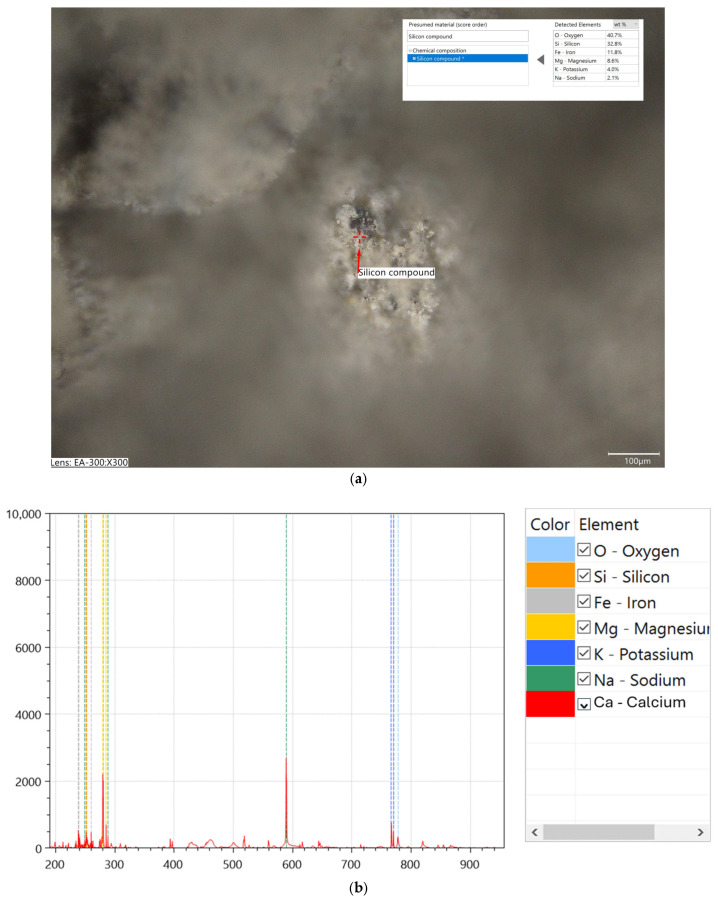
Morphology and chemical composition of chalcedonite at 0/250 µm: (**a**) micrograph with marked location of measurement of the chemical composition (laser-induced breakdown spectroscopy, LIBS) using Keyence EA-300 VHX (magnification: 300×); (**b**) spectra information.

**Figure 4:**
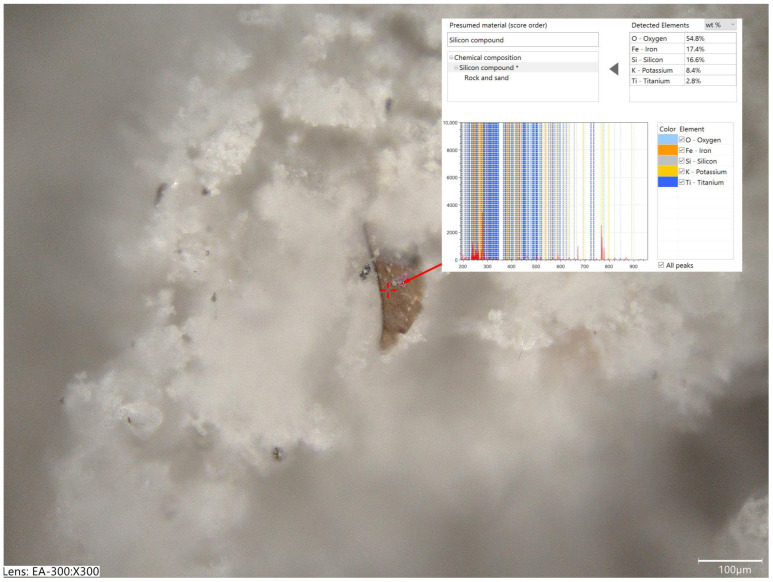
Micrograph with marked location of measurement of the chemical composition (laser-induced breakdown spectroscopy, LIBS) using Keyence EA-300 VHX (magnification: 300×) and spectra information showing the presence of titanium compounds in chalcedonite 0/250 µm.

The simplified elemental chemical analysis based on laser-induced breakdown spectroscopy (LIBS) performed using a Keyence EA-300 VHX analyzer (Keyence Corporation, Osaka, Japan) confirmed that the tested chalcedonite powders contained high contents of silicone compounds (the registered silicon element content during the multi-point measurements was in the range of 18.0–57.1 wt.%). The tests also confirmed the presence of compounds of aluminum, iron, magnesium, potassium, sodium (Figure 3), and titanium (Figure 4).

**Figure 5:**
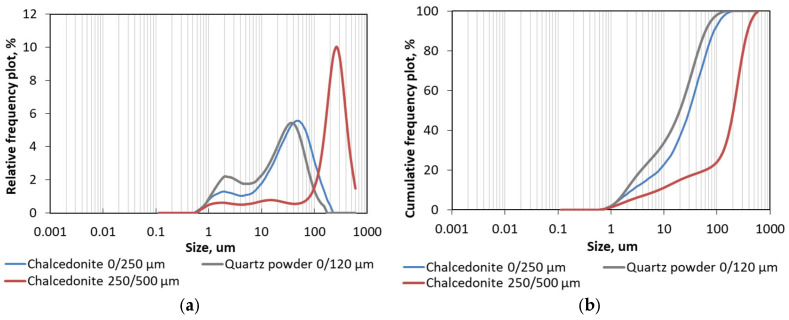
Particle size distribution of quartz powder fraction of 0/120 µm and chalcedonite of fractions of 0/250 µm and 250/500 µm: (**a**) relative frequency plot; (**b**) cumulative frequency plot.

The chalcedonite fillers were then examined for particle size distribution, PSD. It was especially important in the case of the finer fraction of 0/250 µm as it was intended to substitute quartz powder of fraction 0/120 µm. The measurements were performed using a laser diffraction PSD analyzer, Horiba LA-300 (HORIBA, Ltd., Kyoto, Japan). The measurement principles were based on laser diffraction phenomenon and Mie light scattering theory, LST (detailed explanation can be found in [39,40]). The test involved passing laser beams through a 0.1% sodium hexametaphosphate (CAS: 1012-56-8) solution with particles of tested material dispersed by ultrasonics and determining their size (in the range of 0.01–600 μm). Statistical parameters describing the PSD and additional value of the specific surface area, SPA (calculated from the PSD, making an assumption about the spherical shape of the particles), of all tested materials are given in Table 4 and the PSDs in terms of the relative frequency plot and cumulative frequency plot are shown in the Figure 5, which shows the similar grain curve of quartz and finer chalcedonite powder.

The obtained results showed that the finest analyzed fractions, despite the maximal sizes declared by the manufacturers differing by more than two times (i.e., 120 µm and 250 µm), presented much more similar grading than was expected. The median values (19.82 µm and 30.02 µm for quartz powder and chalcedonite powder, respectively) differed only 1.5 times; modes and means differed only 1.3 times. And the specific surface area of chalcedonite (7648 cm^2^/cm^3^) was smaller by 23% than in the case of quartz powder (9973 cm^2^/ cm^3^). In general, the frequency plots adopted very similar characters (Figure 5).

When it comes to the grading of the chalcedonite of the declared fraction of 250/500 µm (or 0.25/0.5 mm), the PSD tests showed that over 65% of the material was actually smaller than 250 µm, while 2.1% of the material presented as larger size than 500 µm (Figure 5b). The mode, i.e., the most common value, was 244.78 µm. In general, the high content of small particles (24% smaller than 100 µm) and the trimodal particle size distribution of this chalcedonite fraction (Figure 5a) meant that its specific surface area was much more developed than expected.

The fact that quartz powder of fraction 0/120 µm and chalcedonite of fraction 0/250 µm showed similar grading confirmed that this chalcedonite fraction can be a good substitute for quartz powder in RPC. However, due to the fact that the second fraction of chalcedonite turned out to be much finer than declared by the manufacturer and it negatively affected the consistency of the concrete, the authors must have redesigned the chalcedonite RPCs’ quantitative compositions in terms of the content of particular chalcedonite fractions and the superplasticizer. This is why, despite the almost identical specific gravity of quartz and chalcedonite, the substitution was not performed 1:1 by weight, thus the reference reactive powder concretes and chalcedonite reactive powder concretes present various quantitative compositions (compare in Table 2). The fact that the fineness of the tested chalcedonite portion was greater than declared by the manufacturer means that when using this material, it is necessary to carefully control the consistency and adjust the dosage of the superplasticizer to obtain the required fluidity.

### 3.3. Reactive Powder Concrete Testing Methods

All reactive powder concretes were characterized in terms of selected technical properties, which included apparent density, flexural strength, compressive strength, capillary action, water absorption, frost resistance, and carbonation resistance (expressed by changes in the flexural and compressive strength of concretes subjected to carbonation).

The flexural strength and compressive strength were determined according to the EN 196-1 European standard, after 28 days of curing (24 h in molds and 27 days in water in laboratory conditions), or after a modified curing procedure including demolding after 24 h, thermal treatment in a water bath at 80 °C for 24 h, and then 5 days in water in laboratory conditions (altogether 7 days) or 26 days (altogether 28 days) of traditional water-curing. Selected specimens were tested after additional exposure to carbonation and cyclic freezing–thawing.

Each flexural strength test was performed using a set of three standard specimens in the shape of prisms (or “beams”) of size 40 mm × 40 mm × 160 mm in the three-point flexural test (using Instron 5567 electromechanical testing machine, Canton, OH, USA). The compressive strength was tested on the same specimens, i.e., prisms’ halves remaining after the flexural test or on cube specimens (using Controls MC66 hydraulic press, CONTROLS S.p.A., Milan, Italy). Apparent density in an air-dry state was determined on the same specimens (mass of the specimens divided by their measured volume) just before the destructive tests.

The results of the flexural strength and compressive strength tests were then the basis for the carbonation assessment. The method described in the EN 12390-12 [41] European standard assumed that after 28-day-long standard curing, the prism specimens were conditioned in laboratory conditions for another 14 days and then exposed to CO_2_ in the carbonation chamber for 70 days. The conditions in the chamber were as follows: CO_2_ concentration of 3 ± 0.5%, relative humidity, RH, of 57 ± 3%, and temperature of 20 ± 2 °C. After 14, 28, 56, and 70 days, the specimens were taken out of the carbonation chamber and a piece of the prism was chipped off and the fracture was treated with phenolphthalein, indicating the depth of carbonation. The method assumed that if after 70 days in the chamber the carbonation depth did not exceed 4 mm, the tested material was characterized by high resistance to carbonation. An additional way of assessing the resistance of concrete to carbonation was the analysis of the flexural strength and compressive strength of specimens exposed to CO_2_ compared with the strength of analogous specimens not subjected to carbonation.

Identical prism specimens of size 40 mm × 40 mm × 160 mm were used for water mass absorption and capillary absorption tests. Those tests were performed to explore the potential of chalcedonite-based RPC for architectural applications, particularly in outdoor environments. Capillary absorption testing was of particular importance, as it provided insight into the material’s resistance to eventual pigment leaching from the mineral matrix.

**Figure 6:**
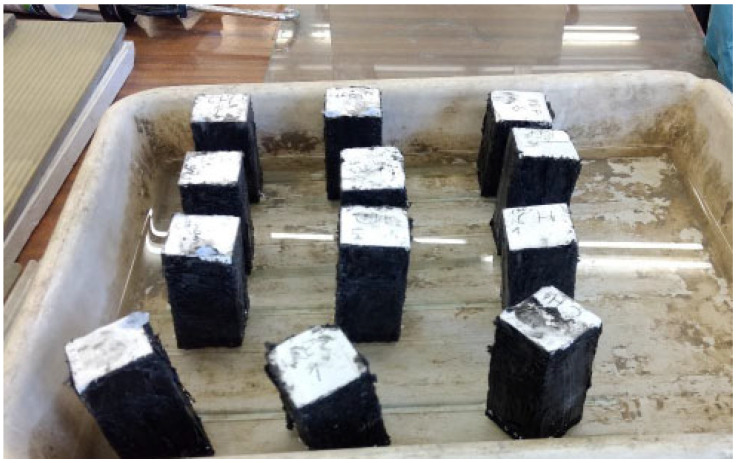
RPC specimens exposed to water (uninsulated fracture side) during capillary action test according to EN 1015-18 European standard method.

The water absorption was determined on the basis of the mass of the specimen saturated with water and the specimen dried to the constant mass, and then the calculation of the percentage of absorbed water to the mass of the dry specimen according to the procedure described in the EN 1015-18 [42] European standard. The capillary absorption test performed according to the same EN 1015-18 European standard required an additional drying of specimens to constant mass and then partial surface sealing: the silicone layer was applied to create a tight coating on the longer sides of the prisms. The specimens were then broken, and the fracture was exposed to distilled water with the possible flow under it (Figure 6).

After 10 and 90 min of absorption and after 24 h of absorption, the mass of the specimens was determined and then the absorption coefficients were calculated according to Formula (1) and expressed in kg/(m^2^·min^0.5^) in the case wherein mortar was not intended as the renovation material or according to Formula (2) and expressed in kg/m^2^ in the case wherein mortar was intended as the renovation material:

C_1_ = 0.1·(M_2_ − M_1_), (1)

C_2_ = 0.625·(M_3_ − M_0_), (2)

where C_1_ and C_2_ are the water absorption coefficients due to capillary action, M_0_ is specimen mass before exposition to water (i.e., dry specimen), M_1_ is the mass of the specimen after 10 min of capillary absorption, M_2_ is the mass of the specimen after 90 min of capillary absorption, and M_3_ is the mass of the specimen after 24 h of capillary absorption.

**Figure 7:**
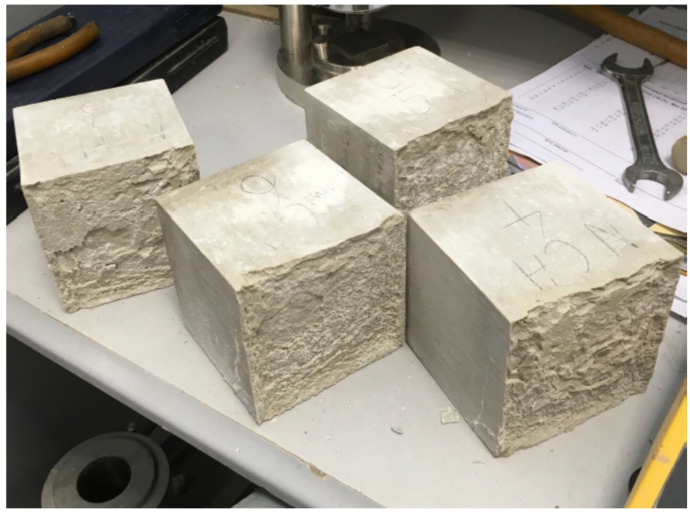
Condition of specimens of chalcedonite RPC (cubes of size 100 mm × 100 mm × 100 mm) after 100 cycles of freezing and thawing.

The test of frost resistance required preparing specimens in the shapes of cubes 100 mm × 100 mm × 100 mm in size. The test was performed according to the “ordinary method” (in water, without chlorides) described in the PN-B-06265 Polish standard [43] (the national supplement to the European standard EN 206 [44]), assuming that the six cube specimens are subjected to 100 cycles of freezing and thawing (1 cycle lasts 9 h and 36 min) and compared to six identical specimens stored in water for the surface condition (Figure 7), in terms of the change in mass and change in compressive strength.

## 4. Results and Discussion

### 4.1. Volumetric Density

**Figure 8:**
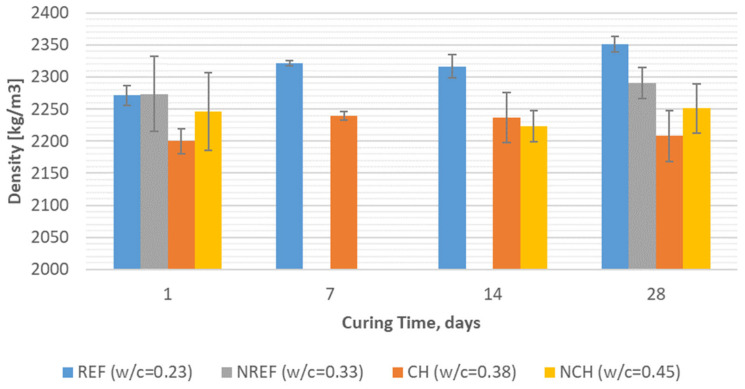
Volumetric density of tested reactive powder concretes: REF/NREF—reference concrete with quartz and silica fume aggregates; CH/NCH—concretes with chalcedonite aggregates.

An examination of the volumetric density results for concretes cured for 28 days (Figure 8 and Table 5) reveals that the density of chalcedonite-based concretes CH and NCH is notably lower—respectively, 2200 kg/m^3^ and 2250 kg/m^3^, less than that of the reference concretes REF and NREF—measured at approximately 2350 kg/m^3^ and 2300 kg/m^3^. This discrepancy is most likely attributable to the porous nature and relatively low intrinsic density of chalcedonite as an aggregate material. Furthermore, taking into consideration the density development during the whole curing process, the density of chalcedonite-based concrete remains relatively stable over time, indicating minimal variation. In contrast, the reference concrete exhibits a marked increase in density, particularly between day 1 and day 7, suggesting a more dynamic hydration and compaction behavior in conventional concrete mixes. There is also a clear trend that as the water/cement ratio increases, the volumetric density of the composite decreases (as a result of increased porosity of the hardened cement paste), and also the coefficient of variance is higher.

Table 5 contains detailed data on the volumetric density of identical 7-day-old concretes subjected and not subjected to thermal treatment. In the case of the quartz-based reference concrete, a higher (by 3.4%) density was noted for the traditionally cured composite (REF) than for the thermally treated concrete (REF^1^). In the case of the chalcedonite-based concrete, the opposite was found: there was a higher (by 4%) density characterized for the composite subjected to an elevated temperature in the early stage of curing (CH^1^). Thus, the effect of changing the curing method on the strength of reactive powder concrete at such an early age could not be clearly determined.

### 4.2. Mechanical Performance

**Figure 9:**
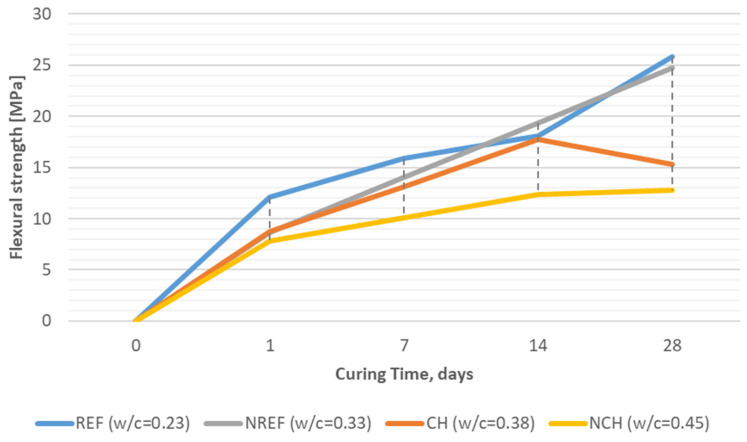
Flexural strength development (average values) of tested reactive powder concretes over time: REF/NREF—reference concrete with quartz and silica fume aggregates; CH/NCH—concretes with chalcedonite aggregates.

**Figure 10:**
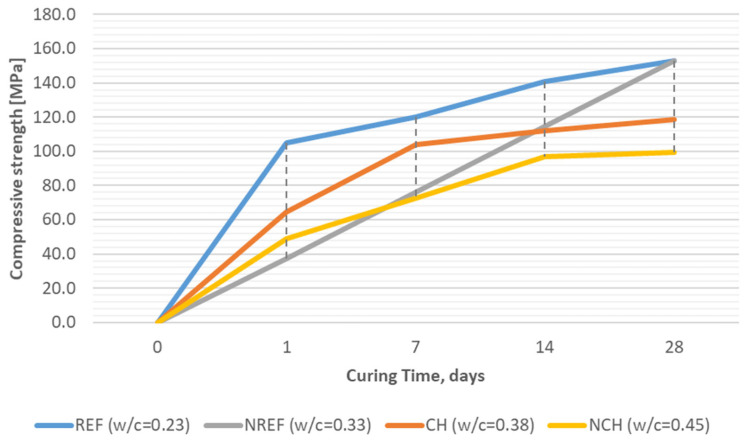
Compressive strength development (average values) of tested reactive powder concretes over time: REF/NREF—reference concrete with quartz and silica fume aggregates; CH/NCH—concretes with chalcedonite aggregates.

As mentioned earlier, the tests of mechanical properties included not only strength determined after 28 days, but also additional measurements at weekly intervals. The authors wanted to examine strength at the initial stage (24 h after casting), but also the development of strength during maturation (after 7 and/or 14 days). An important issue seemed to also be the influence of the curing method on mechanical properties. Figure 9 presents a development of the flexural strength (mean out of three results) in curing time and Figure 10 presents the analogical development of the compressive strength (mean of six results) in curing time for reference concretes (REF, NREF) and for concretes incorporating chalcedonite (CH, NCH).

The reference concrete REF, i.e., the concrete with the lowest w/c ratio equal to 0.23l, after 24 h gained a flexural strength of 12.1 MPa, but after 28 days it obtained 26 MPa. Concrete with a reduced amount of cement and a w/c = 0.33 (NREF) initially obtained only 8.7 MPa, but after 28 days it was 25 MPa. Statistical analysis showed that the obtained results were very repeatable, and the coefficient of variation, CV, was in the range of 2.1–6.5% (the higher CV, 6.0% on average was noted for the concrete with a w/c ratio = 0.33).

In the cases of chalcedonite-based concretes (both with an even higher w/c ratio), the flexural strength development was less intense, initially 7.8–8.8 MPa and steadily growing until the level of 12.8 MPa in the case of the concrete with a w/c = 0.38, and up to 16.1 MPa in the case of the concrete with a w/c = 0.45. Chalcedonite-based concrete with a w/c = 0.38 showed similar homogeneity; the CV did not exceed 6.0%. However, in the case of the concrete with a w/c = 0.45, the coefficient of variation reached values up to 16%.

Nonetheless, flexural strengths of 16 or 26 MPa are very high, close to the level of cement-less polymer concretes (with synthetic resin matrices), especially taking into consideration that ordinary concrete is characterized with a flexural strength usually not exceeding 7–10 MPa [4].

Analysis of the compressive strength test results conducted after 28 days of water-curing showed that in all cases, very-high-performance concrete, VHPC (i.e., with strengths ranging from 100 MPa), was obtained.

The reference concrete with a w/c = 0.23 (REF) reached 105 MPa after just 24 h, and the strength gradually increased up to 153 MPa after 28 days. This concrete also proved to be a homogeneous composite, with a CV in the range of 3.7–6.4%. The second reference concrete with reduced cement content and a w/c = 0.33 (NREF) reached 37.5 MPa after 24 h, but after the full curing period—analogically to the case of the flexural strength test—it achieved the same compressive strength as the REF, i.e., 153 MPa (with a CV not exceeding 4.3%). This means that both quartz-based concretes can be considered as ultra-high-performance concretes, UHPCs, as their compressive strengths exceeded 150 MPa.

As for concretes with chalcedonite fillers, the compressive strength was lower but still very high: in the case of concrete with a w/c = 0.38 (CH), the strength was initially (after 24 h) 63 MPa, after a week it adopted a value of 104.1 MPa, and after 28 days it was 118.5 MPa. The second chalcedonite-based concrete, with the highest water/cement ratio of w/c = 0.45, after a full 28-day-long period of water-curing, developed compressive strength on the level of 100.0 MPa. The results obtained were therefore at a similar level to or higher than those obtained with RPC with other non-standard fillers—e.g., with 4–8% granite dust (compressive strength: 69–92 MPa) [45], 10% steel scoria (compressive strength: 100 MPa) [46], or even RPC additionally reinforced with steel fibers (compressive strength on average: 150 MPa) [47].

**Figure 11:**
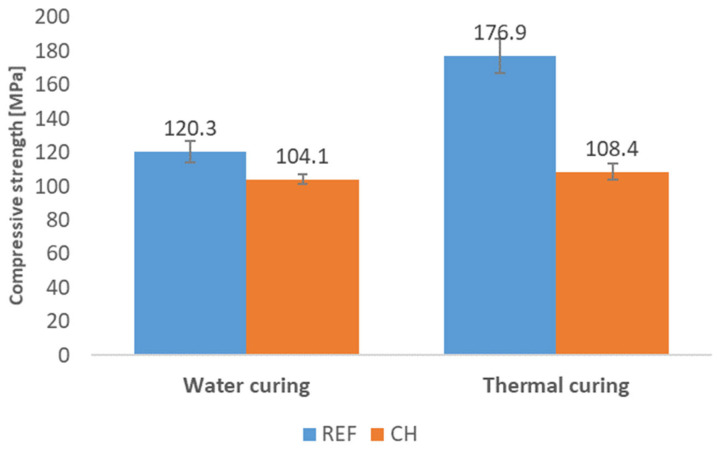
Compressive strength (average values) of reactive powder concretes after 7 days of curing in water or with additional phase of thermal treatment: REF—reference concrete with quartz and silica fume aggregates; CH (w/c = 0.23)—concretes with chalcedonite aggregates (w/c = 0.38).

In Figure 11, there are presented results of compressive strength of concrete REF and CH tested after 7 days of standard water-curing compared to the results obtained for analogical concretes subjected to the thermal treatment (according to the procedure described in Section 3.3., i.e., including demolding after 24 h, thermal treatment in a water bath at 80 °C for 24 h, and then 5 days in water in laboratory conditions—altogether 7 days of curing). Thermal treatment significantly enhanced the strength of the reference concrete—the strength was higher by 56.7 MPa (which corresponds with 47.1%) than the strength of the analogical concrete cured for 7 days in water. In contrast, the concrete incorporating chalcedonite did not exhibit such a significant effect of thermal treatment—the strength of the thermally treated concrete was 108.4 MPa, which is only 4.3 MPa (4.2%) higher in value compared to the analogical concrete cured for 7 days in water. This phenomenon is probably related to the highly porous structure of chalcedonite grains and the influence of this porosity on the hydration process in the contact zone, and perhaps the local lowering of the water/water ratio by pore water. This requires further research, including microstructural studies, which the authors plan to conduct in the next stage.

Composites with chalcedonite were tested at a constant w/c level. Further work is planned to expand the range to include lower and higher w/c values to analyze how the chalcedonite’s intrinsic porosity may influence the effective w/c ratio within the RPC matrix and, thus, how it reflects the relationship between the w/c ratio and mechanical performance.

### 4.3. Water Absorption and Capillary Action

**Figure 12:**
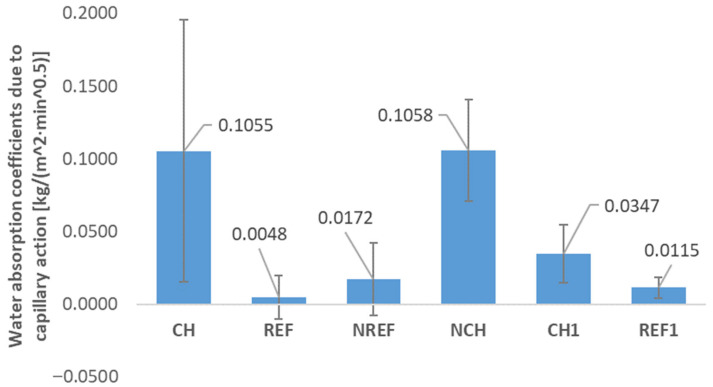
Water absorption coefficients due to the capillary action calculated according to Formula (1) used in the case where mortar was not intended as the renovation material.

**Figure 13:**
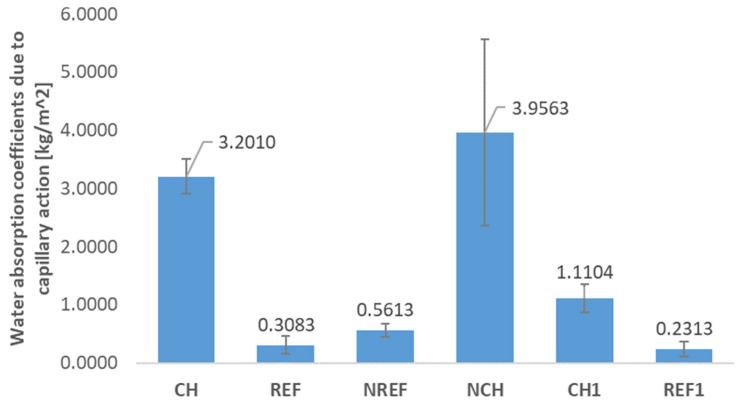
Water absorption coefficients due to the capillary action calculated according to Formula (2) used in case where mortar was intended as the renovation material (REF/NREF—reference concrete with quartz and silica fume aggregates; CH/NCH—concretes with chalcedonite aggregates; REF^1^/CH^1^—concretes subjected to thermal treatment).

**Figure 14:**
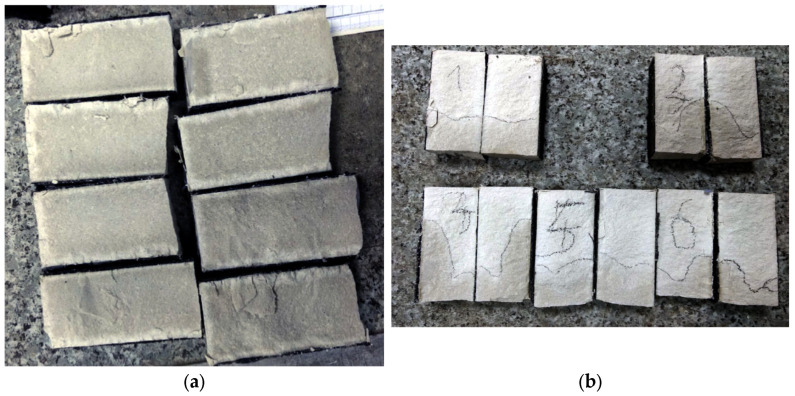
Exemplary fractured RPC specimens: (**a**) specimens of NREF concrete subjected to drying in accordance with the standard method; (**b**) specimens of NCH concrete after the capillary action test and subsequent fracture with the marked capillary action range.

The water absorption coefficients due to the capillary action calculated according to the Formula (1), i.e., the formula used in the case of mortars not intended as the renovation materials (presented in Figure 12), and that calculated according to the Formula (2), i.e., used in the case of mortars intended as the renovation materials (presented in Figure 13), are unfortunately not entirely reliable, as the tests for quartz-based concretes (REF, NREF, and REF^1^) were unsuccessful. Drying according to the EN 1015-18 method did not result in the complete drying of these specimens, and only the outer surfaces dried slightly (an exemplary image of the fractured NREF specimens is shown in Figure 14a). This also affected the results of mass water absorption tests and indicates that the standard method of testing is not applicable for a composite containing fine-grained chalcedonite.

In the cases of chalcedonite-based concretes, the standard drying method was successful. As for the general water absorption of chalcedonite concrete, it turned out to be relatively high at approx. 6% (CH: 6.1%, NCH: 6.8%). The high water absorption is due to the high porosity of chalcedonite rock, and the microstructure of concretes made from such reactive powders. However, it was demonstrated that the thermal treatment enabled us to reduce the water absorption of 7-day-old concrete (CH^1^) to a value of 5.2%, likely by closing the pores during thermal treatment.

Analyzing the results of capillary action tests of concretes containing chalcedonite, it can be noted that they are quite susceptible to capillary rising. Figure 13 shows the height to which the water rose during the capillary test. It is also evident that in the case of thermally treated concrete (CH^1^), the capillary rising was significantly reduced, which suggests that thermal treatment inhibits capillary rising. This can be the effect of the already mentioned potential sealing of the pores during the thermal treatment process.

### 4.4. Durability: Carbonation and Frost Resistance

**Figure 15:**
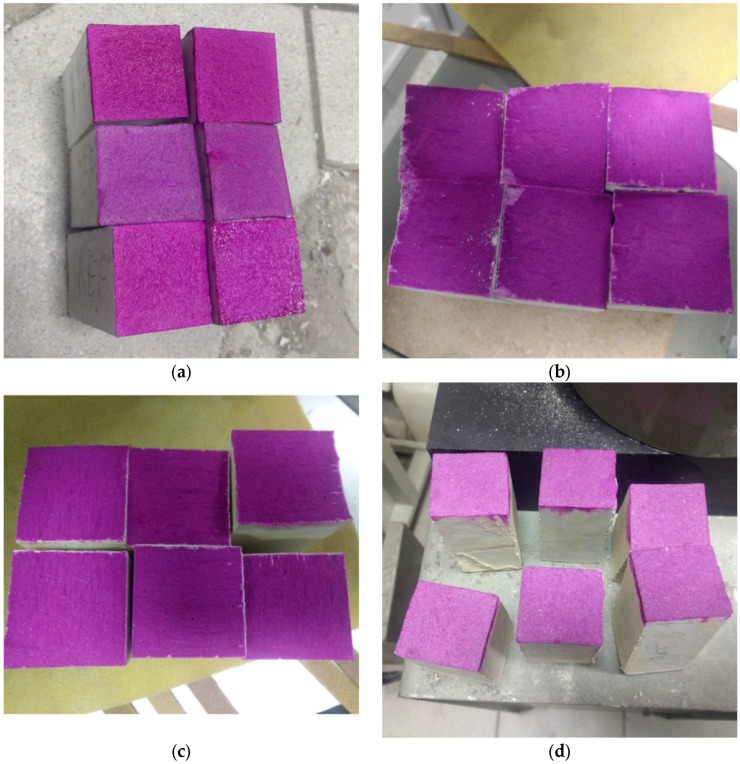
Reactive powder concretes after 70-day-long exposition to CO_2_ and treated with a phenolphthalein indicator showing the depth of carbonation: (**a**) reference quartz-based concrete (REF); (**b**) chalcedonite-based concrete (CH); (**c**) reference quartz-based concrete subjected to thermal curing (REF^1^); (**d**) chalcedonite-based concrete subjected to thermal curing (CH^1^).

Based on observations of fractures of carbonated specimens treated using the phenolphthalein indicator (Figure 15), it can be concluded that all tested BPR concretes—based on quartz (Figure 15a) and on chalcedonite (Figure 15b)—are completely resistant to carbon dioxide. According to the standard, they are highly resistant to carbonation. Also, there was no visible effect of thermal treatment on carbonation; no carbonation was indicated (compare Figure 15c,d).

**Figure 16:**
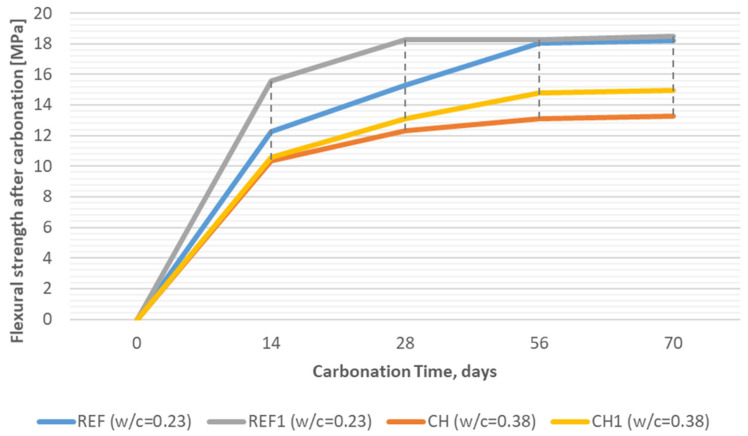
Flexural strength of RPCs tested after 14, 28, 52, and 70 days of exposition to CO_2_ in the carbonation chamber (REF—reference concrete with quartz and silica fume aggregates; CH—concrete with chalcedonite aggregates; REF^1^/CH^1^—concretes subjected to thermal treatment).

**Figure 17:**
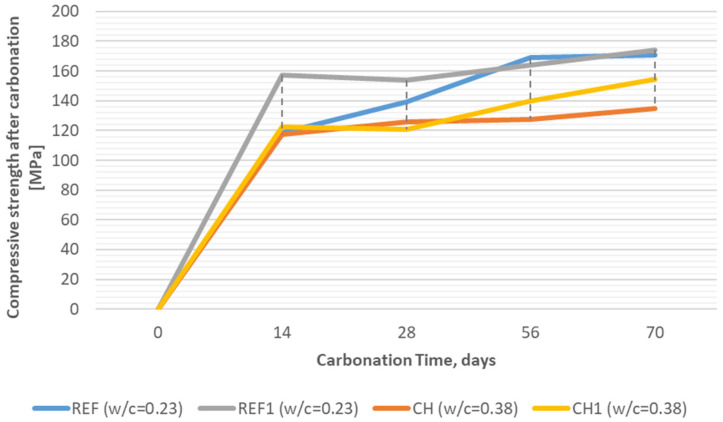
Compressive strength of RPCs tested after 14, 28, 52, and 70 days of exposition to CO_2_ in the carbonation chamber (REF—reference concrete with quartz and silica fume aggregates; CH—concrete with chalcedonite aggregates; REF^1^/CH^1^—concretes subjected to thermal treatment).

However, in an attempt to differentiate the carbonation resistance of individual concretes, the strength of carbonated specimens (that was compared to the strength of the analogical control specimens, not subjected to intensified CO_2_ action) was considered as an additional measure of the durability. Figure 16 presents the flexural strength (mean out of three results) in the time of exposition to CO^2^ in the carbonation chamber, and Figure 17 presents analogical data of the compressive strength (mean of six results) obtained for reference concretes (REF, REF^1^) and for concretes incorporating chalcedonite (CH, CH^1^).

Analyzing the flexural strength results after carbonation, one can conclude that within the scope of the experiment, the strength did not deteriorate with the time of the exposition to CO_2_. The strength of reference quartz-based concretes after 56 days in the chamber obtained a c.a. of 18 MPa, a lower value compared to 28-day-old control specimens not subjected to intensified CO_2_ action, but chalcedonite-based concretes after 70 days in the carbonation chamber obtained the same level of flexural strength as 28-day-old control specimens, i.e., 13 MPa (CV = 1.8%) in the case of concrete with a w/c = 0.38 and 15 MPa (CV = 5.7%) in the case of concrete with a w/c = 0.45.

Analyzing the compressive strength results after carbonation, it can be concluded that the compressive strength is clearly increasing along with the time in the carbonation chamber. This is due to the carbonation products filling the surface pores, making the concrete more resistant to compressive stress [48,49]. The REF and CH^1^ concretes are particularly noteworthy, as they increased their strength by approximately 30 MPa throughout the entire test period. It can be concluded that, for such a long period of exposition (70 days) to an increased level of CO_2_, the effect of carbon dioxide on this type of concrete is positive [50]. It would be worthwhile to conduct a future study of carbonation over a much longer period. The final values of compressive strength obtained after the whole carbonation test duration by reference quartz-based concretes are 170.9 MPa (REF) and 174.3 MPa (REF^1^), which still entitles these concretes to be included in the UHPC group. The final values of compressive strength obtained after the whole carbonation test duration by chalcedonite-based concretes are 134.7 MPa (CH) and 154.6 MPa (CH^1^), which entitles these concretes to be included in the VHPC and UHPC groups even after CO_2_ attack.

The adopted carbonation test time of up to 70 days results from the provisions of EN 12390-12. Longer tests are planned, including those under natural conditions (according to EN 12390-10 [51]) and using other alcacimetric indicators (e.g., thymolphthalein) and the microstructural analysis of carbonation products and their effect on composite properties.

**Figure 18:**
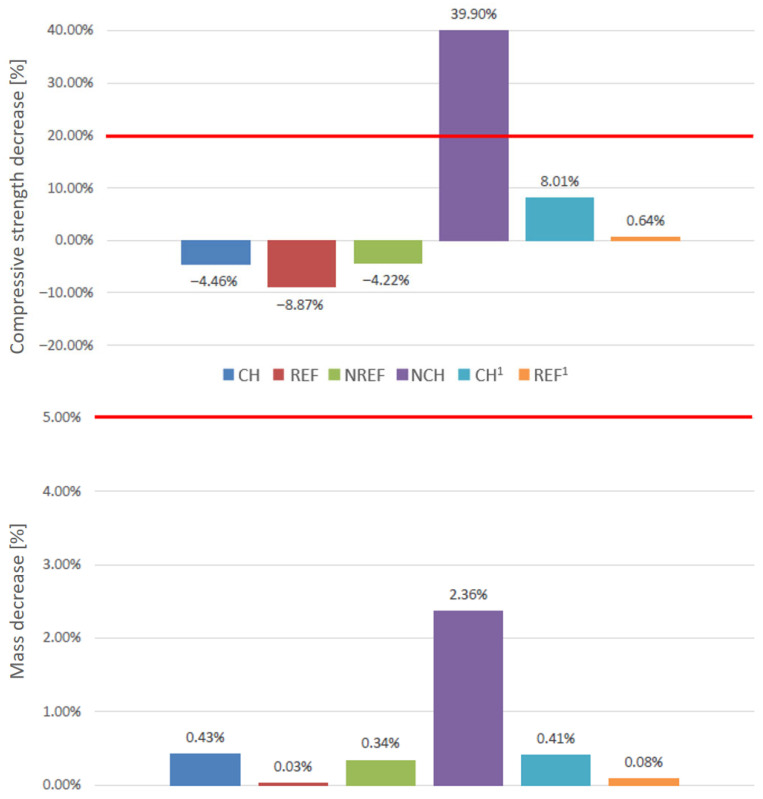
Summary of frost resistance results for average strength loss (upper chart) and mass loss (lower chart).

The results of the frost resistance tests are presented in Figure 18. It is immediately evident that the concrete incorporating chalcedonite with a reduced cement content does not meet the frost resistance criterion (max. 20%) due to a significant loss in compressive strength. In the case of the remaining concretes, the frost resistance requirement is fulfilled, thus these can be considered to be frost resistant.

Analyzing the results obtained for REF, NREF, and CH concretes, it is observed that after the frost resistance test, these concretes exhibit higher compressive strengths than the control specimens (not subjected to cyclic freezing and thawing). This phenomenon may be explained in several ways.

First, it is possible that the concrete is fully resistant to frost, and the observed differences are within the margin of measurement uncertainty. Another explanation could be the formation of microcracks during the test, which allowed water to penetrate deeper into the concrete. Some of the unhydrated cement, which initially acted as a microfiller due to the low water content in the mix, may have undergone hydration after water ingress caused by frost-induced microdamage, thereby increasing the concrete’s strength.

A third explanation involves the relation between pore pressure and the freezing point of water: the smaller the pores, the lower the freezing temperature. The frost resistance of high-performance concrete (HPC) requires understanding the relationship between pore size and the freezing temperature of water within those pores [52]. For instance, macro capillaries (of a characteristic dimension, with R_H_ ranging from 30 μm to 1 mm), meso capillaries (30 μm > R_H_ > 1 μm), and micro capillaries (1 μm > R_H_ > 30 nm) are freezable down to −20 °C, while meso gel pores (30 nm > R_H_ > 1 nm) are not freezable above −23 °C and micro gel pores (R_H_ < 1 nm) are not freezable above −90 °C [53] (after Setzer [54]). Therefore, in the case of HPC, where pore sizes are smaller than in conventional concrete, the freezing of water during standardized frost resistance testing may not occur at all or only to a limited extent. However, further research is necessary to fully explain all aspects of HPC’s resistance to freezing, including the effectiveness of air-entraining admixtures and their side effects on mechanical properties.

## 5. Conclusions

On the basis of the results obtained in the presented research on concretes made from reactive powders, particularly concrete made with chalcedonite instead of standard reactive aggregate, the following conclusions can be formulated:

- The concrete incorporating the chalcedonite aggregate exhibits a lower bulk density compared to the reference concrete with the quartz-based aggregate, which is attributed to the porous nature of chalcedonite. Thermal treatment does not significantly influence the bulk density of the material.
- Thermal treatment did not alter the mechanical strength of chalcedonite-based concretes but it accelerated the curing process. In contrast, the reference concrete demonstrated a substantial increase in strength following thermal treatment compared to standard curing conditions.
- An increase by 0.10 in the water-to-binder ratio in the quartz-based reference concrete formulation (i.e., from 0.33 in the case of the REF concrete to 0.43 in the case of the NREF concrete) did not lead to a significant change in either the mechanical strength or the frost resistance, which proves the possibility of optimizing the quantitative composition of this type of concrete without the need to overstate the w/c ratio or the amount of cement.
- Chalcedonite-based concrete with a reduced cement content—achieved by decreasing the cement-to-aggregate ratio by 12%—exhibited a marked decline in both compressive strength and frost resistance. However, the obtained strength values were very high (compressive strength reaching levels of 100–120 MPa), allowing these composites to be classified as high-performance concretes, HPC.
- All RPC composites showed excellent resistance to carbonation (no shift in the carbonation front inside the concrete was observed) and the mechanical properties were maintained despite the attack of the increased CO_2_ concentration and the cyclic freezing and thawing to which the concretes were subjected.

In summary, chalcedonite, a previously underutilized material classified as a unique rock, has proven its usefulness as a potential aggregate for reactive powder concretes. RPCs designed and manufactured entirely from chalcedonite have proven to be characterized by very high mechanical strength, excellent resistance to carbonation and frost, and therefore very good durability. This opens up highly promising prospects for novel and significantly more advanced applications of this rock compared to its traditional uses. Thus, the unique rock of chalcedonite could serve as the basis for an equally unique composite with excellent mechanical properties, eliminating the need for costly silica fume. Moreover, if the potential production of chalcedonite-based RPCs (either as mixes or precast elements) were carried out in a facility located near the chalcedonite quarry, the resulting concrete would also feature a reduced carbon footprint by minimizing the transportation of alternative aggregates. Nevertheless, for the same reason, it would be worthwhile to investigate similar composites produced with white cement sourced locally, as the cement used in this study was imported from Scandinavia. As part of further research, the authors plan to expand the scope of the study: first of all, the authors intend to investigate chalcedonite-based concretes with varying mix proportions (including different w/c ratios) to broaden the applicability of the current findings to a wider research population, and to incorporate microstructural studies to better examine the binder–chalcedonite aggregate contact zone, which will allow for better interpretation of the results obtained at the macroscale.

## Figures and Tables

**Table 1:** Selected characteristics of the used white cement CEM I 52.5R based on the manufacturer’s data (Aalborg Portland Holding A/S, Aalborg Øst, Denmark).

	Characteristic	Value
Chemical Characteristics [wt.%]	Ignition loss	1.10
	Sulfate content SO_3_	2.10
	Chloride content Cl^−^	≤0.04
	Alkali content Na_2_O_eq_	≤0.30
	Alite (tricalcium silicate) C_3_S	77.00
	Belite (dicalcium silicate) C_2_S	16.00
	Celite (tricalcium aluminate) C_3_A	5.00
	Brownmillerite (tetracalcium aluminatferrite) C_4_AF	1.00
Physical properties	Initial setting time [min]	120
	Specific gravity [kg/m^3^]	3130
	Bulk density [kg/m^3^]	1100
	Fines (Blain SPA) [m^2^/kg]	400
	Stability of volume [mm]	0.5
Compressive strength [MPa]	After 1 day	24
	After 2 days	44
	After 7 days	60
	After 28 days	72

**Table 2:** Compositions of tested reactive powder concretes per 1 m^3^; REF/NREF—reference concrete with regular quartz and silica fume aggregates; CH/NCH—concretes with chalcedonite aggregates.

No/Code	Cement	Chalcedonite		Quartz		Silica f. 0/120 µm	Water	Admix.	w/c
		0/250 µm	250/500 µm	0/120 µm	250/500 µm				
	[kg]							[%]	[kg/kg]
1/REF	903	-	-	271	767	181	210	2.2	0.23
2/REF ^1^									
3/CH	810	548	548	-	-	-	305	2.2	0.38
4/CH ^1^									
5/NREF	838	-	-	251	711	168	276	2.2	0.33
6/NCH	712	576	576	-	-	-	320	2.6	0.45

^1^ Composite subjected to thermal treatment while curing.

**Table 3:** Chemical composition (expressed by oxides, [wt.%]) of chalcedonite from Teofilów deposits according to the manufacturer’s data (CRUSIL, Inowłódz, Poland) [38] and according to [22].

Compound	Content ^1^ [%] [33]	Content ^1^ [%] [22]
SiO_2_	94.35–99.54	94.35–99.60
Al_2_O_3_	0.40–3.69	1.56–2.70
Fe_2_O_3_	0.12–0.49	0.25–0.36
CaO	0.01–0.10	0.90–1.20
MgO	0.01–0.04	0.06–0.33
K_2_O	0.06–0.42	K_2_O + Na_2_O: 0.22–0.27
Na_2_O	0.06–0.42	
TiO_2_	0.06–0.42	-

^1^ by wt.

**Table 4:** Statistical parameters describing the particle size distribution and specific surface area of quartz powder fraction of 0/120 µm and chalcedonite of fractions 0/250 µm and 250/500 µm.

Parameter	Quartz Powder 0/120	Chalcedonite 0/250	Chalcedonite 250/500
D_min_ [µm]	0.67	0.58	0.67
D_10_ [µm]	2.19	2.60	8.82
D_50_ (median) [µm]	19.82	30.02	201.85
D_m_ (mean) [µm]	26.46	39.13	201.08
D_90_ [µm]	67.52	88.58	394.24
Mode [µm]	36.55	47.99	244.78
SPA ^1^ [cm^2^/cm^3^]	9973	7648	3779

^1^ Calculated from the distribution, making an assumption about the spherical shape of the particles.

**Table 5:** Apparent density of tested reactive powder concretes (REF/NREF—reference concrete with quartz and silica fume aggregates; CH/NCH—concretes with chalcedonite aggregates; REF^1^/CH^1^—concretes subjected to thermal treatment).

No/Code	Age [Days]	Density [kg/m^3^]	SD [kg/m^3^]	CV [%]	w/c [kg/kg]
1a/REF	7	2322	4	0.2	0.23
1b/REF	28	2351	13	0.5	
2/REF^1^	7	2244	18	0.8	
3a/CH	7	2239	7	0.3	0.38
3b/CH	28	2208	40	1.8	
4/CH^1^	7	2326	16	0.6	
5/NREF	28	2291	24	1.1	0.33
6/NCH	28	2251	38	1.7	0.45

## Data Availability

The original contributions presented in this study are included in the article. Further inquiries can be directed to the corresponding author.

## Abbreviations

The following abbreviations are used in this manuscript:

CEM	Cement
CV	Coefficient of variation
D_min_	Minimal diameter
D_10_	Diameter/size below which 10% of all particles are found
D_50_	Median diameter
D_m_	Mean diameter
D_90_	Diameter/size below which 90% of all particles are found
EN	European Norm (Standard)
HPC	High-performance concrete
LIBS	Laser-induced breakdown spectroscopy
LST	Light scattering theory
OC	Ordinary concrete
OPC	Ordinary Portland cement
PSD	Particle size distribution
SCM	Supplementary cementing material
SD	Standard deviation
SPA	Specific (surface) area
R_H_	Characteristic dimension of the pore in cement paste
RPC	Reactive powder concrete
VHPC	Very-high-performance concrete
UHPC	Ultra-high-performance concrete
